# Manual Therapy Exerts Local Anti-Inflammatory Effects Through Neutrophil Clearance

**DOI:** 10.1155/2024/5556042

**Published:** 2024-11-05

**Authors:** Hongwen Liu, Shiguo Yuan, Kai Zheng, Gaofeng Liu, Junhua Li, Baofei Ye, Yangkun Wang, Li Yin, Yikai Li

**Affiliations:** ^1^School of Traditional Chinese Medicine, Southern Medical University, Guangzhou, Guangdong Province, China; ^2^Clinical Research Center, Department of Orthopaedic, Panzhihua Central Hospital, Panzhihua, Sichuan Province, China; ^3^Department of Orthopaedic, Hainan Traditional Chinese Medicine Hospital, Hainan Medical University, Haikou, Hainan Province, China; ^4^Department of Orthopaedic, Guangdong Provincial Hospital of Chinese Medicine, Hainan Hospital, Guangzhou University of Chinese Medicine, Haikou, Hainan Province, China; ^5^The Third Affiliated Hospital, Southern Medical University, Guangzhou, Guangdong Province, China

**Keywords:** inflammation, manual therapy, neutrophils, S100A9, skeletal muscle

## Abstract

**Background:** Manual therapy (MT) has been widely used in China to treat local tissue inflammation for a long time. However, there is a lack of scientific evidence for using MT in anti-inflammatory therapy, and its anti-inflammatory mechanism needs further clarification.

**Methods:** We utilized MT to treat cardiotoxin (CTX) injury-induced skeletal muscle inflammation in C57BL6/J mice. We analyzed the underlying mechanism by integrating single-cell RNA sequencing (scRNA-seq) with molecular techniques. Hematoxylin and eosin (H&E) and immunohistochemical (IHC) staining were used to assess skeletal muscle inflammation and muscle fiber cross-sectional area (CSA). scRNA-seq, immunofluorescence, and western blot were performed to determine cellular and molecular outcome changes.

**Results:** Compared with CTX injury-induced skeletal muscle inflammatory mice, MT intervention significantly reduced proinflammatory cytokines interleukin (IL)-1*β*, IL-6, and tumor necrosis factor alpha (TNF-*α*) expression levels; scRNA-seq detected that neutrophil numbers and activity were maximum proportions increased in injured skeletal muscle among macrophage, T cells, B cells, endothelial cells, fast muscle cells, fibroblasts, and skeletal muscle satellite cells; and S100A9 gene expression was supreme in neutrophils. However, after treatment with MT, S100A9 protein expression and the numbers and activity of Ly6g+/Mpo+ neutrophils were significantly inhibited, thus reducing the inflammatory cytokine levels and exerting an anti-inflammatory effect by early clearing neutrophils.

**Conclusion:** MT can mitigate localized inflammation induced by injured skeletal muscle, achieved by decreasing S100A9 protein expression and clearing neutrophils in mice, which may help advance therapeutic strategies for skeletal muscle localized inflammation.

**Summary**

This study provides compelling evidence suggesting a potential link between manual therapy (MT) and anti-inflammatory immune responses. Specifically, our findings highlight that:

• Neutrophils are the most abundant and active cells, playing a predominant role in the early stages of inflammation rather than macrophages.
• S100A9 may serve as a marker for activating neutrophils in local inflammation.
• Early administration of MT can reduce proinflammatory cytokines interleukin (IL)-1*β*, IL-6, and tumor necrosis factor alpha (TNF-*α*) levels by inhibiting S100A9 protein expression and clearing neutrophils in inflamed skeletal muscles.

## 1. Introduction

Skeletal muscle inflammation caused by traumatic accidents, inappropriate exercise, and a sedentary lifestyle has become a significant public health issue [1, 2]. Moderate inflammation is beneficial to the recovery of injured muscles. However, excessive inflammation is detrimental to the repair of muscle and functional recovery, which may ultimately limit activities of daily living, diminish quality of life, and contribute to poor rehabilitative outcomes in skeletal muscle injury individuals [3]. Skeletal muscle inflammation is a typical localized inflammation. The primary treatment modalities include oral nonsteroidal anti-inflammatory drugs (NSAIDs), hormones, and diuretics. Nevertheless, this is a systemic treatment with poor targeting and many adverse effects. Other treatments include local icing and topical medications, but manual therapy (MT) is the most popular treatment among individuals [4–6].

MT, also referred to as tuina or massage in China, encompasses the application of mechanical pressure to injured tissue. It is widely recognized in alternative and complementary medicine for its ability to rehabilitate the musculoskeletal tissue [7, 8]. This potential benefit is likely attributed to various effects, such as enhanced blood circulation, reduced reactive oxygen species (ROS) levels, and increased mitochondrial biogenesis [9]. Studies on muscle regeneration have indirectly indicated that MT may partially reduce the levels of proinflammatory cytokines [10, 11]. However, the study's results could be more comprehensive, leading to low credibility. Many scholars believe that MT should not be used for treatment in the early stages of a muscle injury because it may aggravate the injury and the anti-inflammatory mechanisms of MT remain largely unexplored. In particular, the target immune cell is still unclear—is it a macrophage, a T cell, a B cell, or a neutrophil?

Therefore, the present work aims to evaluate whether MT can effectively mitigate the localized inflammation induced by skeletal muscle injury, specifically through regulating immune cell response. In our study, neutrophils were screened as MT's most critical target cell cluster to relieve early localized inflammation. We demonstrated that MT can improve the inflammatory microenvironment in the skeletal muscle by inhibiting the activation of S100A9 protein and clearance of neutrophils. Thus, our findings provide experimental and theoretical foundations for potential anti-inflammatory mechanisms of MT.

## 2. Materials and Methods

All methods adhered to the Animal Research: Reporting of In Vivo Experiments (ARRIVEs) guidelines. The study's design and experimental protocols were formulated by the guide for the care and use of laboratory animals, as outlined by the US National Institute of Health (NIH; Publication No. 8023, revised 1978). Approval for the study was obtained from the Institutional Animal Care and Use Committee at Southern Medical University (Approval No. L2023063).

### 2.1. Animal

In vivo experiments were conducted on C57BL6 male mice, with a weight ranging from 20 to 25 g and an age of 8–9 weeks, obtained from the Animal Experimentation Center of Southern Medical University. The mice were housed in standard conditions, including a 22 ± 2°C temperature, a 12-h light/dark cycle, and relative humidity between 50% and 60%. Additionally, they had ad libitum access to food.

### 2.2. Model Establishment

Following the established methods, a skeletal muscle inflammation model was induced in mice's tibialis anterior (TA) muscles [1, 2, 12]. Briefly, mice were anesthetized with 2% isoflurane, and 10 μL of cardiotoxin (CTX, 10 μg/mL) was injected into the right TA muscle during anesthesia. Subsequently, the mice were randomly assigned to four groups: normal mice (*n* = 6), sham mice (phosphate-buffered saline (PBS), *n* = 6), model mice (CTX, *n* = 6), and MT mice (MT, *n* = 6). The normal group received no injection, while the sham group received a 10 μL injection of PBS into the TA muscle.

### 2.3. MT Intervention

The specific MT intervention, guided by both published research and our prior investigations [2, 4, 6], was administered as follows:

1. The intervention commenced 24 h postinjury and continued for 7 consecutive days, involving 5-min sessions every 10–12 h.
2. Mice were familiarized with the experimenter's handling for 10 min before the intervention.
3. A pressure tester was utilized to apply pressure and massage the midpoint of the right TA muscle, providing real-time feedback on pressure levels and enabling adjustments as needed by the operator (Figure S1).
4. The pressure tester maintained a consistent stimulation pressure of 0.5 N and a frequency of 1 Hz.

Groups not receiving MT underwent daily handling procedures akin to those of the MT group to control (CON) for potential stress-related effects. After treatment, the mice were returned to their cages. Muscular strength was assessed daily through a grip test throughout the experiment. On the eighth day, mice were anesthetized, and blood samples were collected via heart puncture exsanguination. Serum was obtained following centrifugation (2200 g, 10 min). TA muscle samples were collected and stored in a −80°C ultralow temperature refrigerator (Haier, Qingdao, China) for protein extraction or preserved in 4% paraformaldehyde for histological analysis.

### 2.4. Histopathological Staining

The right TA muscle was collected, preserved in 4% paraformaldehyde, encased in paraffin, and sliced into 4–6 μm sections. These sections underwent heating at 60°C for 2 h, followed by dewaxing with xylene, gradual rehydration using alcohol, and rinsing with PBS. Hematoxylin and eosin (H&E) staining was employed to assess the muscle fibers' cross-sectional area (CSA). All histological evaluations and analyses were conducted in a blinded fashion.

### 2.5. Immunohistochemical (IHC) Staining

For IHC staining, slides underwent antigen retrieval by boiling in the buffer for 30 min, followed by blocking endogenous peroxidases with 3% H_2_O_2_ for 10 min. Tissue sections were treated with 5% bovine serum albumin (BSA) for 60 min at room temperature (RT) to prevent nonspecific binding. Subsequently, they were exposed to primary antibodies interleukin (IL)-1*β* (1:500, #ab283818, Abcam) and IL-6 (1:100, #ab300582, Abcam) overnight at 4°C. After a 30-min rewarming period, sections were washed with PBS and then incubated with horseradish peroxidase (HRP)-conjugated secondary antibodies for 20 min at RT. Following this, sections were stained with diaminobenzidine (DAB; #ENZ-ACC105-0200, Enzo Life Sciences) and counterstained with H&E staining. An isotype CON was included as a negative CON. Photomicrographs were captured using a BX43 microscope (Olympus, Tokyo, Japan), and IHC images were analyzed utilizing ImageJ software.

### 2.6. Immunofluorescence

As previously outlined, TA muscle tissue slides were subjected to heating and deparaffinization. Following this, they were permeabilized with 0.5% phosphate-buffered saline with Tween 20 (PBST) for 45 min at RT before exposure to primary antibodies (Ly6g, 1:1000, #ab25377, Abcam; Mpo, 1:1000, #ab252131, Abcam) overnight at 4°C without light. After three washes in PBST, sections were treated with Cy3-conjugated antirat immunoglobulin G (IgG; 1:2000, #A0516, Beyotime Biotechnology) or Alexa Fluor 594-conjugated antirabbit secondary antibodies (1:1000, #ab150092, Abcam) for 60 min in darkness. Subsequently, the sections were washed thrice with PBST, and the nuclei were stained with 4′,6-diamidino-2-phenylindole (DAPI; #1002, Beyotime Biotechnology) for 2 min. Imaging of TA muscle tissue sections was performed using a BX43 microscope (Olympus, Tokyo, Japan). Three fields were randomly chosen from each section to determine the average fluorescence intensity of the target protein using ImageJ software.

### 2.7. Western Blot

Total protein was extracted from TA muscle tissue using radioimmunoprecipitation assay (RIPA) buffer. The protein concentration was determined utilizing a Bicinchoninic Acid Protein Assay Kit. Then, proteins were separated via sodium dodecyl sulfate-polyacrylamide gel electrophoresis (SDS-PAGE) and transferred onto polyvinylidene fluoride membranes. These membranes were then treated with 5% nonfat milk to block nonspecific binding and incubated overnight at 4°C with primary antibodies (IL-1*β*, 1:1000, #ab283818, Abcam; IL-6, 1:1000, #ab300582, Abcam; tumor necrosis factor alpha (TNF-*α*), 1:1000, #ab307164, Abcam; S100A9, 1:1000, #ab242945, Abcam; and actin, 1:5000, #ab179467, Abcam) diluted in PBS. After washing with Tris-buffered saline with Tween 20 (TBST), the membranes were exposed to secondary antibodies at RT for 60 min. Protein bands were visualized using the ChemiDoc MP system (Bio-Rad, California, USA) coupled with an enhanced chemiluminescence (ECL) kit (#E423-01, Vazyme) and subsequently analyzed using ImageJ software. The experiments were conducted thrice to ensure reliability.

### 2.8. Single-Cell RNA Sequencing (scRNA-Seq) Data Processing

Our experimental approach incorporated scRNA-seq data from the GEO dataset, focusing on CTX-induced inflammation in the TA muscles of C57BL6/J mice. This experimental group contrasted with scRNA-seq data obtained from healthy C57BL6/J mice, constituting the CON group. Specifically, we identified GSM3614992 and GSM3614993 as the CON group, while GSM4831162 and GSM4831163 represented the skeletal muscle inflammation group (CTX) within the dataset. These datasets underwent a standard scRNA-seq analysis using the Illumina NovaSeq platform and were subsequently processed using CellRanger software (10x Genomics) to generate the feature-barcode gene expression matrix [13, 14].

For subsequent analyses, we utilized the Seurat R package (version 4.3.0) to perform principal component analysis (PCA) and Uniform Manifold Approximation and Projection (UMAP) analysis. Cells containing fewer than 200 genes, more than 7500 genes, or over 20% mitochondrial genes, were excluded, resulting in the analysis of 13,337 filtered cells. Gene expression normalization was conducted using the “LogNormalize” method, followed by scaling. PCA identified significant principal components (PCs), with the *p*-value distribution visualized using the JackStraw and ScoreJackStraw functions. Batch correction used the “Harmony” R package (version 0.1.1) to mitigate any batch effects hindering downstream analysis. Subsequently, 10 PCs were selected for UMAP analysis. The FindClusters function categorized cells into 14 distinct clusters with a resolution of 0.5. Differential expression analysis for each cluster was performed using the FindAllMarkers function with a logfc.threshold of 0.25. Cell types were identified based on the differentially expressed genes (DEGs) within each cluster and cross-verified manually with findings from previous studies.

### 2.9. Inflammation-Related Genes' (IRGs) Area Under the Curve (AUC) Score

Using the GSEA database (https://www.gsea-msigdb.org/), we determined each cluster's IRGs' scores. The AUCell R package (version 1.12.0) facilitated this analysis by scoring pathways in each cell based on gene set enrichment analysis. Gene expression rankings were created for each cell to estimate the proportion of highly expressed genes within the gene set. Cells with more expressed genes within the set exhibited higher AUC values. The “AUCell_exploreThresholds” function was utilized to establish the threshold for identifying active cells in the gene set. Finally, the AUC scores of individual cells were depicted on the UMAP embedding using the ggplot2 R package (version 3.4.2) to visualize active clusters.

### 2.10. Statistical Analyses

Statistical analyses were conducted using GraphPad Prism software (version 7, San Diego, California), with results presented as mean ± standard deviation. We initially assessed sample normality using the Shapiro–Wilk test. For data that followed a normal distribution, Students' *t*-tests and analysis of variance (ANOVA) were employed to compare parametric data between two conditions and among multiple conditions, respectively. Post hoc pairwise comparisons in ANOVA were performed using Tukey's test. In nonparametric or nonnormally distributed data, we used the Mann–Whitney *U* test to compare two groups and the Kruskal–Wallis test for multiple groups, followed by Dunn's post hoc tests. Additionally, a two-way ANOVA followed by Bonferroni's test was conducted for analyses involving two independent variables. Statistical significance was considered at *p*  < 0.05, with significance levels indicated as *⁣*^*∗*^*p* < 0.05, *⁣*^*∗∗*^*p* < 0.01, and *⁣*^*∗∗∗*^*p* < 0.001.

## 3. Results

### 3.1. MT Promptly Relieves Localized Inflammation in Skeletal Muscle

**Figure 1:**
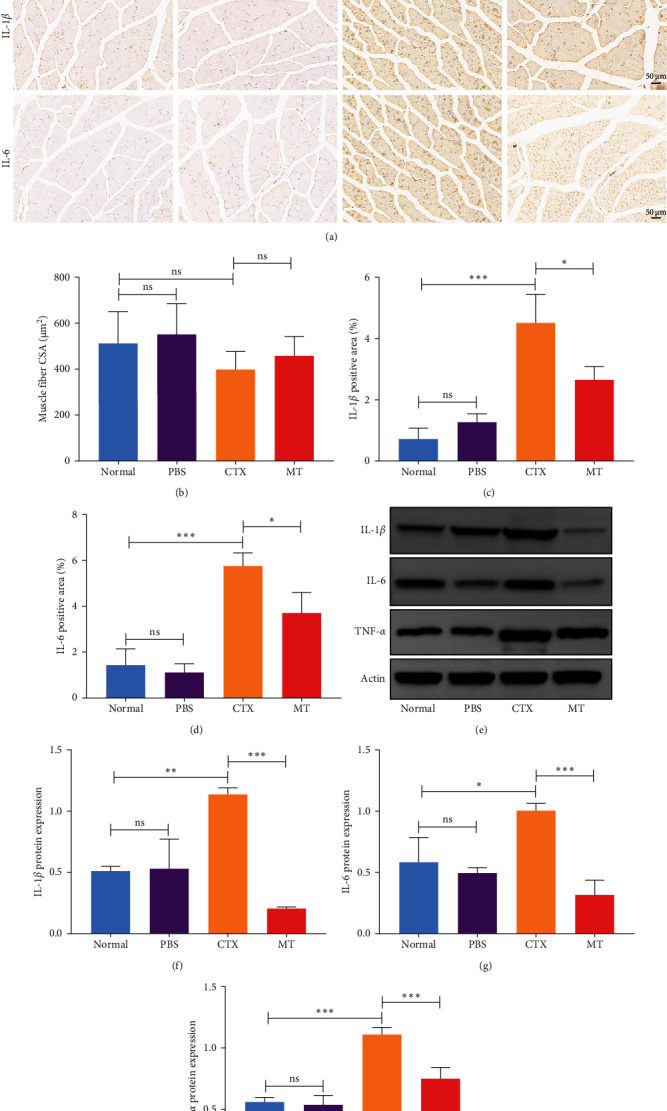
MT relieves localized inflammation in the skeletal muscle. (A) Representative H&E and immunohistochemistry staining (IL-1*β* and IL-6) images of cross-histological sections of TA muscle. Scale bars = 50 μm. (B) Quantification of muscle fiber CSAs. (C, D) Percentage of IL-1*β* and IL-6 positive regions (appearing brown in immunohistochemistry staining in A). (E, F, G, H) Western blot analysis shows the relative protein expression of IL-1*β*, IL-6, and TNF-*α*. CSA, cross-sectional area; CTX, cardiotoxin; H&E, hematoxylin and eosin; IL, interleukin; MT, manual therapy; ns, not significant; PBS, phosphate-buffered saline; TA, tibialis anterior; TNF-*α*, tumor necrosis factor alpha. *⁣*^*∗*^*p* < 0.05, *⁣*^*∗∗*^*p* < 0.01, and *⁣*^*∗∗∗*^*p* < 0.001.

To investigate whether MT affects local immune responses in treated skeletal tissues, we quantified the levels of proinflammatory cytokines IL-1*β*, IL-6, and TNF-*α* in the TA muscle undergoing MT. We also compared the CSA between the MT-treated and the CON groups. Seven days after CTX-induced inflammation in the TA muscle, no differences in CSA between the groups were observed (Figure 1A,B), suggesting that significant atrophy of the TA muscle did not occur. However, the TA muscle tissue exhibited infiltration by many inflammatory cells. Although this infiltration did not disrupt the structure of skeletal muscle fibers, a substantial increase in the expression of IL-1*β* and IL-6 was observed.

Conversely, after MT treatment, the expression of IL-1*β* and IL-6 was significantly lower than that in the CTX group (Figure 1C,D). In line with these findings, IL-1*β* and IL-6 protein expression decreased in the TA muscle after MT treatment (Figure 1E–G). Moreover, TNF-*α* protein expression decreased in the MT group (Figure 1H). These results indicate that CTX successfully induces inflammation in skeletal muscle, and MT intervention can alleviate localized inflammation in skeletal muscle.

### 3.2. Neutrophils Are the Primary Effector Cells of Localized Inflammation in Skeletal Muscle

**Figure 2:**
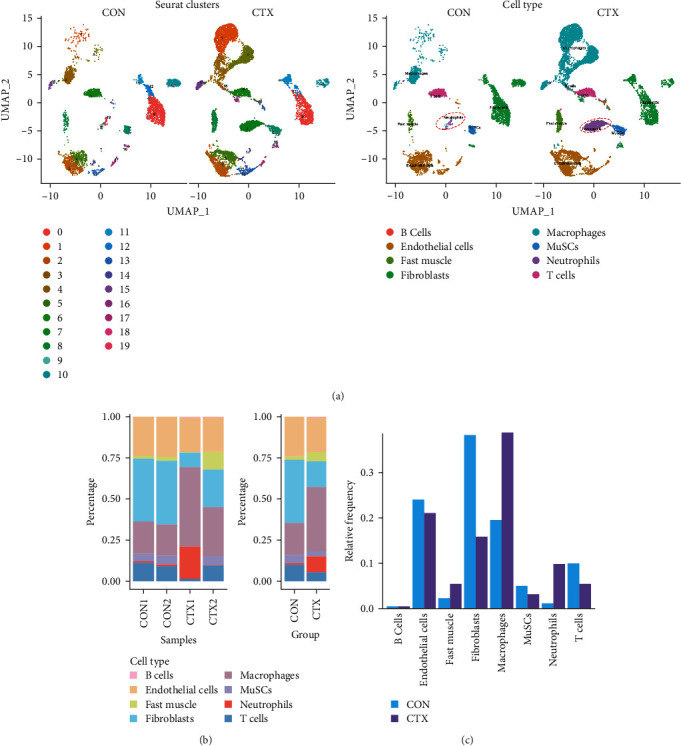
Single-cell transcriptomic characterization and alterations in cell clusters of injured skeletal muscle. (A) UMAP plot presenting all sequenced cells based on cell type, with distinct cell types represented by unique colors, and UMAP projection of CON and muscle fibrosis (CTX) groups; neutrophils are denoted with ellipses. (B, C) Cluster distribution in each sample and group; neutrophils show a maximum increased percentage in the CTX group. CON, control; CTX, cardiotoxin; MuSCs, muscle satellite cells; UMAP, Uniform Manifold Approximation and Projection.

Subsequently, we examined whether changes in proinflammatory cytokines were associated with alterations in specific immune cell populations within inflamed muscle tissue. To achieve this, we analyzed scRNA-seq data obtained from C57BL6/J mice following CTX injection-induced skeletal muscle inflammation and compared it with data from normal mice. Integrating scRNA-seq data from the GEO dataset enabled the characterization of cell populations. After filtration, 8944 cells from injured (CTX) mice and 4393 cells from CON mice remained for analysis. A total of 19 cell types were identified, resulting in eight distinct cell clusters visualized using UMAP plots based on cell type–specific marker genes (Figure 2A). These clusters comprised B cells (CD19+, MS4A1+, and CD22+), endothelial cells (PECAM1+ and CD34+), fast muscle cells (MYH1+), fibroblasts (PDGFRA+), macrophages (CSF1R+ and CD68+), skeletal muscle satellite cells (MuSCs) (Myod1+ and Pax7+), neutrophils (S100a8+, S100a9+, CD14+, and ITGAM+), and T cells (CD3D+, CD3E+, and CD8A+).

Cell counts were significantly altered within the neutrophils, macrophage, and fast muscle clusters. Notably, these clusters exhibited a marked increase in the CTX group compared to the CON group. The neutrophil cluster, in particular, showed the highest percentage increase in the CTX group (Figure 2B,C). Table S1 provides the number of cells in each cluster, respectively. These findings indicate that neutrophils are the most numerous and active cells, predominantly influencing the level of inflammation. Therefore, we infer that neutrophils play a critical role in modulating inflammation levels in skeletal muscles. Subsequent analyses focused primarily on neutrophils.

### 3.3. IRGs Are Primarily Activated in Neutrophils

**Figure 3:**
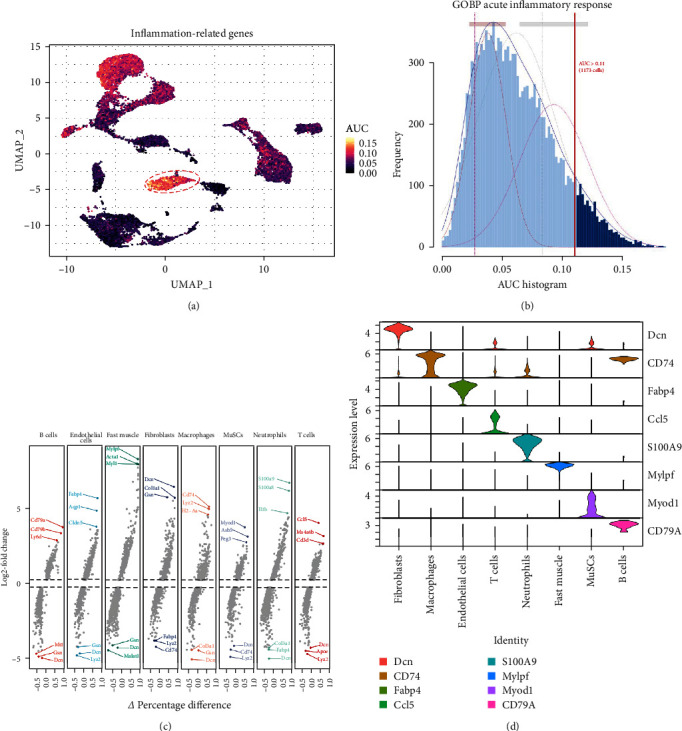
IRGs AUC scores and differentially expressed genes (DEGs) in injured skeletal muscle. (A) UMAP plots of IRGs AUC scores in all clusters. Neutrophils exhibit the largest gene expression and AUC values. (B) AUC scores for IRGs, with a threshold set at 0.12. (C) High expression of the top three genes and low expression of the bottom three genes in cell clusters. (D) Violin plots illustrate cell-type marker gene distributions in each cluster using density curves. Each violin plot's width corresponds to the cells' frequency with the relevant gene expression level. AUC, area under the curve; GOBP, Gene Ontology biological process; IRGs, inflammation-related genes; MuSCs, muscle satellite cells; UMAP, Uniform Manifold Approximation and Projection.

To further elucidate the role of activated neutrophils, we analyzed the activation of 135 IRGs across eight cell clusters identified by scRNA-seq. These clusters included B cells, endothelial cells, fast muscle cells, fibroblasts, macrophages, MuSCs, neutrophils, and T cells. IRGs specific to skeletal muscle were obtained from the GSEA database (https://www.gsea-msigdb.org/), which compiles IRGs from published research, resulting in a list of 135 IRGs. We utilized the AUCell R package to evaluate the activity of IRGs in each cell cluster. Cells expressing more genes exhibited higher AUC values, predominantly observed in neutrophils and macrophages (Figure 3A,B). Notably, neutrophils attained higher AUC values. Gene expression analysis revealed that S100A9, S100A8, and IL-1*β* were the three most highly expressed genes in neutrophils, with S100A9 being the most DEG (Figure 3C,D). These findings indicate significant activation of IRGs in inflamed skeletal muscle tissue, with the majority of expression occurring in neutrophils, suggesting that S100A9 may serve as a marker gene for neutrophil activation in local inflammation.

### 3.4. Neutrophil-Related Genes Are Predominantly Expressed in the Inflammatory Skeletal Muscle

**Figure 4:**
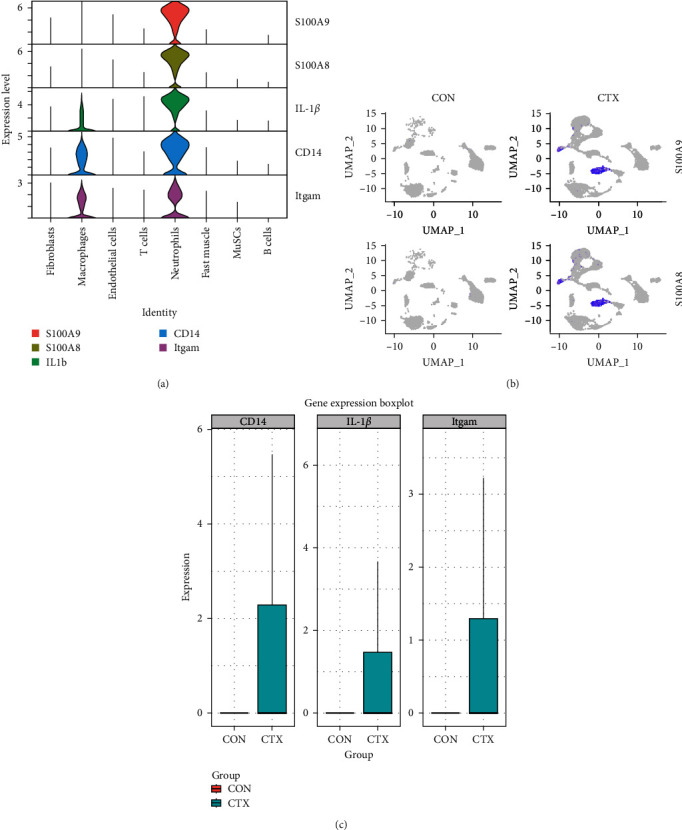
Neutrophil-related gene expression in injured skeletal muscle. (A) S100A9, S100A8, CD14, IL-1*β*, and Itgam expression in each cluster. (B) S100A9 and S100A8 expression levels in the CON and CTX injured groups. (C) CD14, IL-1*β*, and Itgam expression levels in the CON and CTX injured groups. CON, control; CTX, cardiotoxin; IL, interleukin; MuSCs, muscle satellite cells.

To analyze the differential expression of neutrophil-related genes in inflamed and healthy muscle tissue, we examined the expression levels of S100A9, S100A8, IL-1*β*, CD14, and Itgam between the CTX and CON groups. We confirmed that S100A9, S100A8, IL-1*β*, CD14, and Itgam are predominantly expressed in neutrophils rather than other cell types (Figure 4A). Compared to the CON group, all of these genes showed upregulation in the inflamed muscle tissue of the CTX group (Figure 4B,C). These findings suggest that in inflamed muscle tissue, neutrophil function is fully activated, and the most DEG, S100A9, may serve as a marker gene for neutrophil activation in local inflammation.

### 3.5. MT Exerts a Local Anti-Inflammatory Effect by Rapidly Clearing Neutrophils

**Figure 5:**
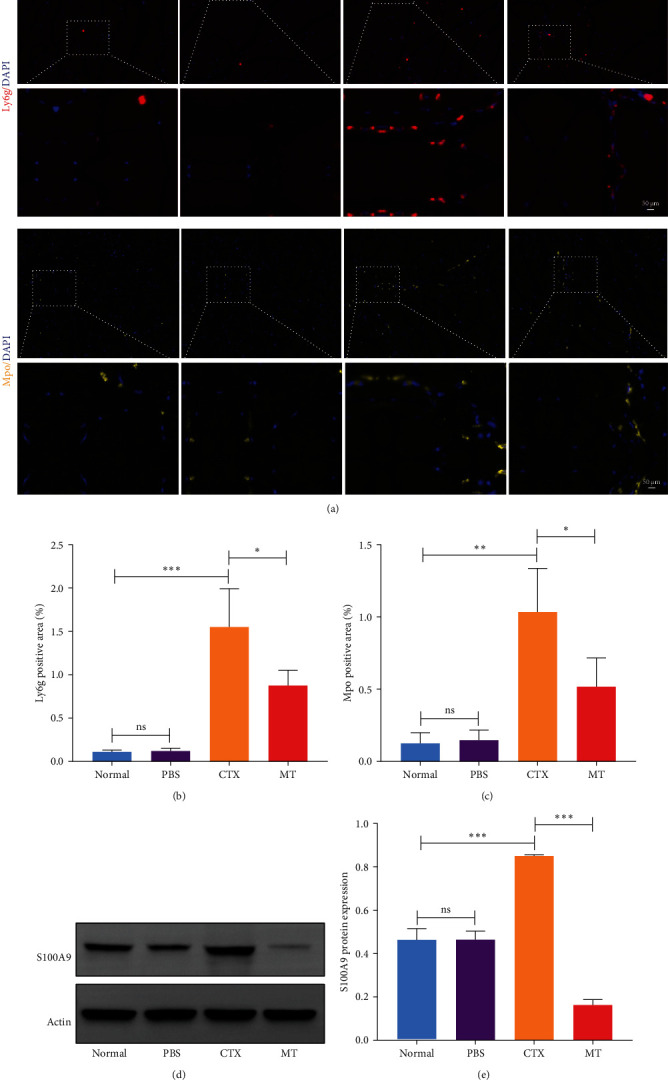
Characterization of neutrophils. (A) Representative immunofluorescence images of cross-histological sections of TA muscle. (B, C) Percentage of Ly6g+ and Mpo+ positive area (appearing as red and yellow in immunofluorescence in A); nuclear staining was performed with DAPI (blue). (D, E) Western blot analysis shows the relative protein expression of S100A9. CTX, cardiotoxin; DAPI, 4′,6-diamidino-2-phenylindole; MT, manual therapy; ns, not significant; PBS, phosphate-buffered saline; TA, tibialis anterior. Scale bars = 50 μm. *⁣*^*∗*^*p* < 0.05, *⁣*^*∗∗*^*p* < 0.01, and *⁣*^*∗∗∗*^*p* < 0.001.

Next, we evaluated the impact of MT regulation on neutrophils and S100A9 in inflamed muscle tissue. Similar to the outcomes observed in proinflammatory cytokine treatment, MT significantly reduced the populations of cells expressing Ly6g+ and myeloperoxidase (Mpo+), two classic markers of neutrophils, in TA muscle tissue compared to the untreated group. Analysis through immunofluorescence imaging revealed that Ly6g+ neutrophils and their peroxidase enzyme, Mpo, were widely distributed in the inflamed muscles without MT. In contrast, they were confined to limited areas in MT-treated muscles (Figure 5A). Moreover, the intensity of positive Ly6g+ and Mpo+ expression levels was notably decreased compared to the CTX group (Figure 5B,C). Consistent with the results of immunofluorescence imaging analysis, MT-treated muscles exhibited a significant reduction in S100A9 protein expression after 7 days of treatment compared to untreated muscles (Figure 5D,E). These findings corroborate the outcomes of the analysis of the proinflammatory cytokine, indicating that MT modulates localized immune responses in inflammatory TA muscle, mainly through its effects on neutrophils and S100A9 during the early stages of the immune cell response.

## 4. Discussion

Inflammation serves as a fundamental defense mechanism in the human body. A complex network involving various immune cells and molecules regulates inflammation, working to eliminate harmful substances and safeguard the body from both internal and external threats [15, 16]. However, disruptions in this network, such as excessive or prolonged inflammation, can result in further tissue damage [12, 17]. Our study provides compelling evidence suggesting a potential link between MT and anti-inflammatory immune responses. Specifically, our findings highlight that (1) neutrophils are the most abundant and active cells, playing a predominant role in the early stages of inflammation rather than macrophages; (2) S100A9 may serve as a marker for activating neutrophils in local inflammation; and (3) early administration of MT can reduce proinflammatory cytokines IL-1*β*, IL-6, and TNF-*α* levels by inhibiting S100A9 protein expression and clearing neutrophils in inflamed skeletal muscles. To our knowledge, this study represents the first empirical evidence suggesting that early application of MT can mitigate inflammation levels following skeletal muscle injury, offering new insights into the potential use of MT as a therapeutic approach against local inflammation.

Skeletal muscle consists of diverse cell types that can detect and respond to mechanical stresses through mechanotransduction [18, 19]. Recent studies have highlighted the immunomodulatory effects of MT on maintaining skeletal muscle balance [2, 6, 9]. Our current research investigated how MT influences the immune microenvironment during skeletal muscle inflammation following injury, with a particular emphasis on neutrophil. Previous research has indicated that applying cyclic mechanical loading immediately after eccentric exercise-induced injury reduced the number of inflammatory macrophages and neutrophils in the muscle [2]. However, this trend may be context-specific, as previous work observed load-dependent increases in macrophage populations within the healthy TA muscle of rats subjected to mechanical loading. This study used a severe skeletal muscle injury model combining ischemic surgery with myotoxin injection. Severe skeletal muscle injury usually accompanies systemic inflammation, and severe broad inflammation is not amenable to mechanical loading treatments. Our study used MT for localized inflammation treatment and observed positive anti-inflammatory effects, which align better with clinical application and lifestyle needs. Additionally, laboratory experiments have demonstrated that mechanical loading prompts macrophages to adopt an anti-inflammatory state [10, 20, 21]. Interestingly, applying controlled cyclic compressive loading to uninjured muscle induces the expression of immune-related genes and attracts CD68+ macrophages and CD163+ cells in a load-dependent manner. Although many current studies on MT and inflammation have focused on macrophages, our findings reveal that neutrophils are the most abundant and active cells, particularly in the early stages of inflammation. Hence, further research should pay more attention to neutrophils.

In the initial phase of inflammation, anti-inflammatory efforts mainly aim to regulate the quantity and behavior of neutrophils rather than macrophages. Neutrophils naturally migrate to damaged muscle tissue in response to acute injury, which is crucial in shaping the subsequent immune response [22]. However, an excessive activation of neutrophils following muscle injuries can exacerbate the initial damage and hinder effective muscle repair [1]. Neutrophils secrete various growth factors like fibroblast growth factor (FGF), hepatocyte growth factor (HGF), insulin-like growth factor-1 (IGF-1), vascular endothelial growth factor (VEGF), and transforming growth factor (TGF)-*β*1, as well as inflammatory cytokines and chemokines such as TNF-*α*, IL-6, CCL17, and CCL2. During the early phase, proinflammatory factor secretions like TNF-*α*, IL-1*β*, IL-6, interferon (IFN)-g, CXCL1, and CXCL4 are prevalent[23]. As monocytes enter the muscle, they encounter IFN-g and TNF-*α*, which prompt their differentiation into macrophages [24]. In our current investigation, we observed that the early growth rate of neutrophils exceeded that of macrophages, and IRGs were predominantly expressed in neutrophils with greater intensity and AUC value than macrophages. This underscores the significance of promptly regulating neutrophil quantity and activity. With early MT intervention, inflammation triggered by skeletal muscle injury could be swiftly managed, leading to significant reductions in the expression levels of proinflammatory factors like TNF-*α*, IL-1*β*, and IL-6, along with effective CON of neutrophil numbers and activity.

Neutrophils contain calprotectin, a heterodimer protein known as S100A9, which has various proinflammatory effects [25, 26]. S100A9 makes up to 45% of all neutrophil cytoplasmic proteins [17]. While it is also found in monocytes and dendritic cells, its levels are much lower in these cells. Inflammation and infection models often show increased levels of S100A9 [27]. It acts as an alarmin, signaling the host about potential danger, and its levels rise notably in conditions like sepsis and severe COVID-19 infection [28, 29]. Studies have demonstrated that calprotectin can enhance the migration and accumulation of neutrophils at sites of inflammation. However, its role in skeletal muscle inflammation remains unclear [25]. Our study reveals that under conditions of skeletal muscle inflammation, S100A9 is the most highly expressed gene by neutrophils. This suggests that it may contribute to neutrophil activation and the accumulation of cytokines in injured skeletal muscle. Following MT treatment, the expression of S100A9 protein decreased along with neutrophil numbers and activity. These findings imply that S100A9 plays a significant proinflammatory role in localized inflammation and could serve as a marker and potential target for protecting against inflammation and muscle damage associated with the disease.

Our study recognizes several limitations. Firstly, the quantification and standardization of MT pose challenges in the current research. Previous studies have identified an effective range of loading force for MT interventions in mice, typically between 0.15 and 0.6 N; forces below 0.15 N showed no therapeutic impact, while forces above 0.6 N caused damage [2]. Based on these findings and prior research, we chose a therapeutic loading force 0.5 N for MT. However, the effects of other mechanical parameters, such as loading frequency, application point, and number of cycles, require further investigation [4]. Secondly, although we used a skeletal muscle inflammation model induced by CTX injection, additional studies employing other clinically relevant inflammation models, such as those resulting from immunological disorders or infections, are needed to improve clinical translation [18]. Thirdly, while our study demonstrated that MT could partly alleviate muscle inflammation by early clearance of neutrophils, leading to reduced expression of the S100A9 protein, the mechanistic link underlying this effect requires further clarification.

## 5. Conclusion

This study's results validate MT's potential in reducing localized inflammation caused by skeletal muscle injury. This effect is attributed to decreased S100A9 protein expression and the clearance of neutrophils in the injured muscle. These findings offer promising implications for clinical applications, particularly in enhancing therapeutic approaches for individuals with skeletal muscle inflammation. However, a deeper understanding of the mechanistic connection between MT and S100A9 protein is needed to comprehend its clinical significance fully.

## Data Availability

Single-cell RNA-seq counts and raw data have been posted on Gene Expression Omnibus (https://www.ncbi.nlm.nih.gov/gds/, GSE126834 and GSE159500), and the other datasets used and/or analyzed during the current study are available from the corresponding author on reasonable request. The code is available from the corresponding author on reasonable request.

## Supporting Information

Supporting Information Figure S1. Diagram of MT intervention. Figure S2. The original, uncropped western blot images of IL-1*β*. Figure S3. The original, uncropped western blot images of IL-6. Figure S4. The original, uncropped western blot images of TNF-*α*. Figure S5. The original, uncropped western blot images of S100A9. Figure S6. The original, uncropped western blot images of ACTIN1. Figure S7. The original, uncropped western blot images of ACTIN2. Table S1. Cell numbers of each cluster.

## References

[1] Nakka K., Hachmer S., Mokhtari Z. (2022). JMJD3 Activated Hyaluronan Synthesis Drives Muscle Regeneration in an Inflammatory Environment. *Science*.

[2] Seo B. R., Payne C. J., McNamara S. L. (2022). Skeletal Muscle Regeneration With Robotic Actuation-Mediated Clearance of Neutrophils. *Science Translational Medicine*.

[3] Tidball J. G. (2017). Regulation of Muscle Growth and Regeneration by the Immune System. *Nature Reviews Immunology*.

[4] Cezar C. A., Roche E. T., Vandenburgh H. H., Duda G. N., Walsh C. J., Mooney D. J. (2016). Biologic-Free Mechanically Induced Muscle Regeneration. *Proceedings of the National Academy of Sciences*.

[5] Duarte F. C. K., Funabashi M., Starmer D. (2022). Effects of Distinct Force Magnitude of Spinal Manipulative Therapy on Blood Biomarkers of Inflammation: A Proof of Principle Study in Healthy Young Adults. *Journal of Manipulative and Physiological Therapeutics*.

[6] Yao C., Ren J., Huang R. (2022). Transcriptome Profiling of microRNAs Reveals Potential Mechanisms of Manual Therapy Alleviating Neuropathic Pain Through microRNA-547-3p-Mediated Map4k4/NF-*κ*b Signaling Pathway. *Journal of Neuroinflammation*.

[7] Aguilar-Agon K. W., Capel A. J., Fleming J. W., Player D. J., Martin N. R. W., Lewis M. P. (2021). Mechanical Loading of Tissue Engineered Skeletal Muscle Prevents Dexamethasone Induced Myotube Atrophy. *Journal of Muscle Research and Cell Motility*.

[8] Bernard C., Zavoriti A., Pucelle Q., Chazaud B., Gondin J. (2022). Role of Macrophages During Skeletal Muscle Regeneration and Hypertrophy—Implications for Immunomodulatory Strategies. *Physiological Reports*.

[9] Barbe M. F., Harris M. Y., Cruz G. E. (2021). Key Indicators of Repetitive Overuse-Induced Neuromuscular Inflammation and Fibrosis Are Prevented by Manual Therapy in a Rat Model. *BMC Musculoskeletal Disorders*.

[10] Dziki J. L., Giglio R. M., Sicari B. M. (2018). The Effect of Mechanical Loading Upon Extracellular Matrix Bioscaffold-Mediated Skeletal Muscle Remodeling. *Tissue Engineering Part A*.

[11] Loerakker S., Stekelenburg A., Strijkers G. J. (2010). Temporal Effects of Mechanical Loading on Deformation-Induced Damage in Skeletal Muscle Tissue. *Annals of Biomedical Engineering*.

[12] Cheng N., Liu C., Li Y. (2020). MicroRNA-223-3p Promotes Skeletal Muscle Regeneration by Regulating Inflammation in Mice. *Journal of Biological Chemistry*.

[13] Ma P., Amemiya H. M., He L. L. (2023). Bacterial Droplet-Based Single-Cell RNA-Seq Reveals Antibiotic-Associated Heterogeneous Cellular States. *Cell*.

[14] Katzenelenbogen Y., Sheban F., Yalin A. (2020). Coupled scRNA-Seq and Intracellular Protein Activity Reveal an Immunosuppressive Role of TREM2 in Cancer. *Cell*.

[15] Costamagna D., Costelli P., Sampaolesi M., Penna F. (2015). Role of Inflammation in Muscle Homeostasis and Myogenesis. *Mediators of Inflammation*.

[16] Pinniger G. J., Lavin T., Bakker A. J. (2012). Skeletal Muscle Weakness Caused by Carrageenan-Induced Inflammation. *Muscle & Nerve*.

[17] Wang S., Song R., Wang Z., Jing Z., Wang S., Ma J. (2018). S100A8/A9 in Inflammation. *Frontiers in Immunology*.

[18] Liu H., Yuan S., Liu G. (2024). Satellite Cell-Derived Exosomes: A Novel Approach to Alleviate Skeletal Muscle Atrophy and Fibrosis. *Advanced Biology*.

[19] Sosa P., Alcalde-Estévez E., Asenjo-Bueno A. (2021). Aging-Related Hyperphosphatemia Impairs Myogenic Differentiation and Enhances Fibrosis in Skeletal Muscle. *Journal of Cachexia, Sarcopenia and Muscle*.

[20] Adams S., Wuescher L. M., Worth R., Yildirim-Ayan E. (2019). Mechano-Immunomodulation: Mechanoresponsive Changes in Macrophage Activity and Polarization. *Annals of Biomedical Engineering*.

[21] Ji X., Yuan X., Ma L. (2020). Mesenchymal Stem Cell-Loaded Thermosensitive Hydroxypropyl Chitin Hydrogel Combined With a Three-Dimensional-Printed Poly(*ε*-Caprolactone)/Nano-Hydroxyapatite Scaffold to Repair Bone Defects via Osteogenesis, Angiogenesis and Immunomodulation. *Theranostics*.

[22] Toumi H., F’guyer S., Best T. M. (2006). The Role of Neutrophils in Injury and Repair Following Muscle Stretch. *Journal of Anatomy*.

[23] Liu H., Yuan S., Zheng K. (2024). IL-17 Signaling Pathway: A Potential Therapeutic Target for Reducing Skeletal Muscle Inflammation. *Cytokine*.

[24] Torres-Ruiz J., Alcalá-Carmona B., Alejandre-Aguilar R., Gómez-Martín D. (2023). Inflammatory Myopathies and beyond: The Dual Role of Neutrophils in Muscle Damage and Regeneration. *Frontiers in Immunology*.

[25] Ding Z., Du F., Averitt V R. G. (2021). Targeting S100A9 Reduces Neutrophil Recruitment, Inflammation and Lung Damage in Abdominal Sepsis. *International Journal of Molecular Sciences*.

[26] Liu Q., Lei X., Cao Z. (2022). TRPM8 Deficiency Attenuates Liver Fibrosis through S100A9-HNF4*α* Signaling. *Cell & Bioscience*.

[27] Shabani F., Farasat A., Mahdavi M., Gheibi N. (2018). Calprotectin (S100A8/S100A9): A Key Protein between Inflammation and Cancer. *Inflammation Research*.

[28] Chen L., Long X., Xu Q. (2020). Elevated Serum Levels of S100A8/A9 and HMGB1 at Hospital Admission Are Correlated With Inferior Clinical Outcomes in COVID-19 Patients. *Cellular & Molecular Immunology*.

[29] Dubois C., Marcé D., Faivre V. (2019). High Plasma Level of S100A8/S100A9 and S100A12 at Admission Indicates a Higher Risk of Death in Septic Shock Patients. *Scientific Reports*.

