# Supplementary material for: Manual Therapy Exerts Local Anti-Inflammatory Effects Through Neutrophil Clearance
Source: J Immunol Res. 2024 Nov 5;2024:5556042. doi: 10.1155/2024/5556042 (PMC11557174; doi:10.1155/2024/5556042)
Supplement: Supporting Information — Figure S1. Diagram of MT intervention. Figure S2. The original, uncropped western blot images of IL-1β. Figure S3. The original, uncropped western blot images of IL-6. Figure S4. The original, uncropped western blot images of TNF-α. Figure S5. The original, uncropped western blot images of S100A9. Figure S6. The original, uncropped western blot images of ACTIN1. Figure S7. The original, uncropped western blot images of ACTIN2. Table S1. Cell numbers of each cluster. [file 5556042.f1.doc]

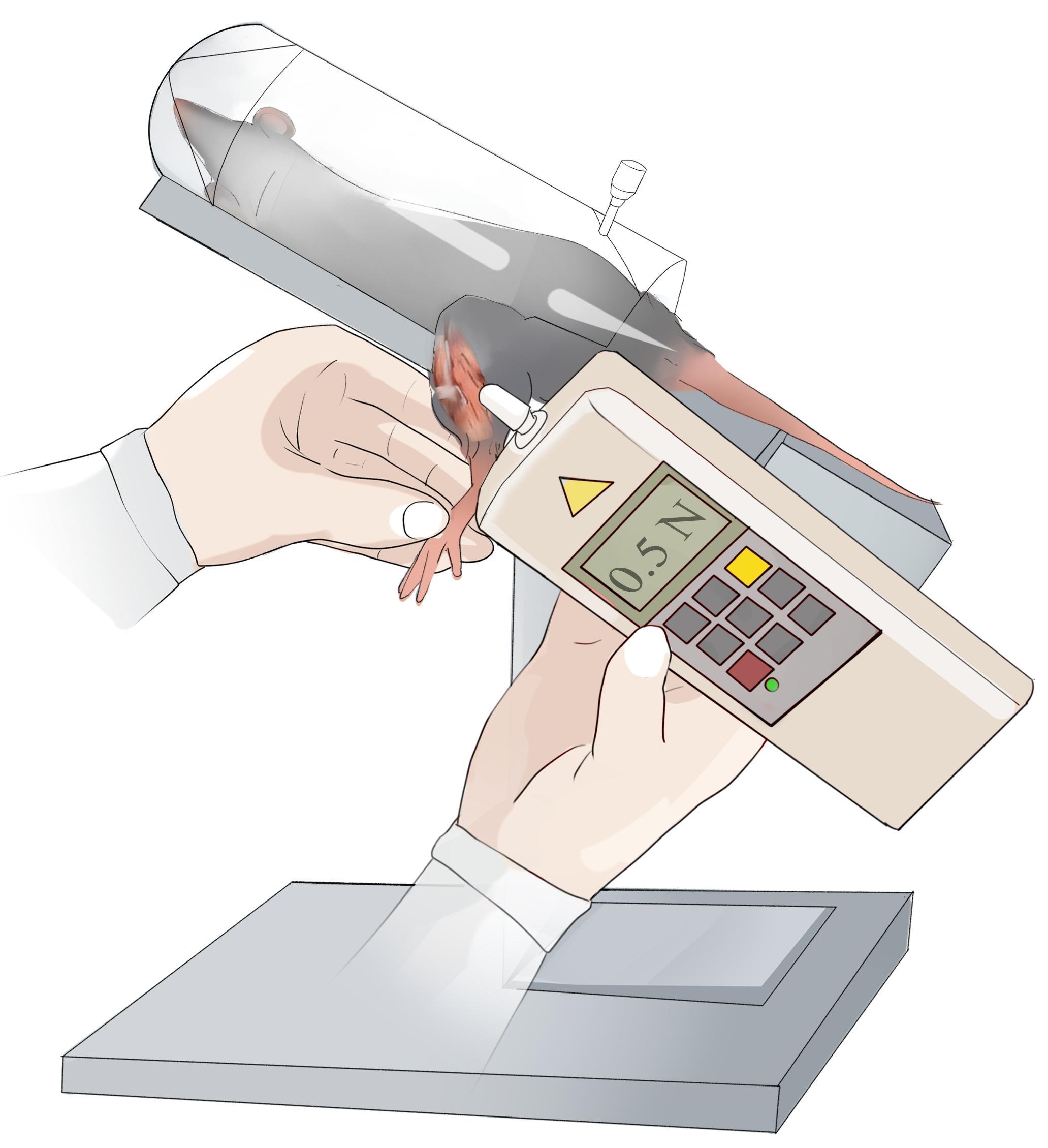


**Supplemental fig. 1 Diagram of MT intervention.** MT, manual therapy.


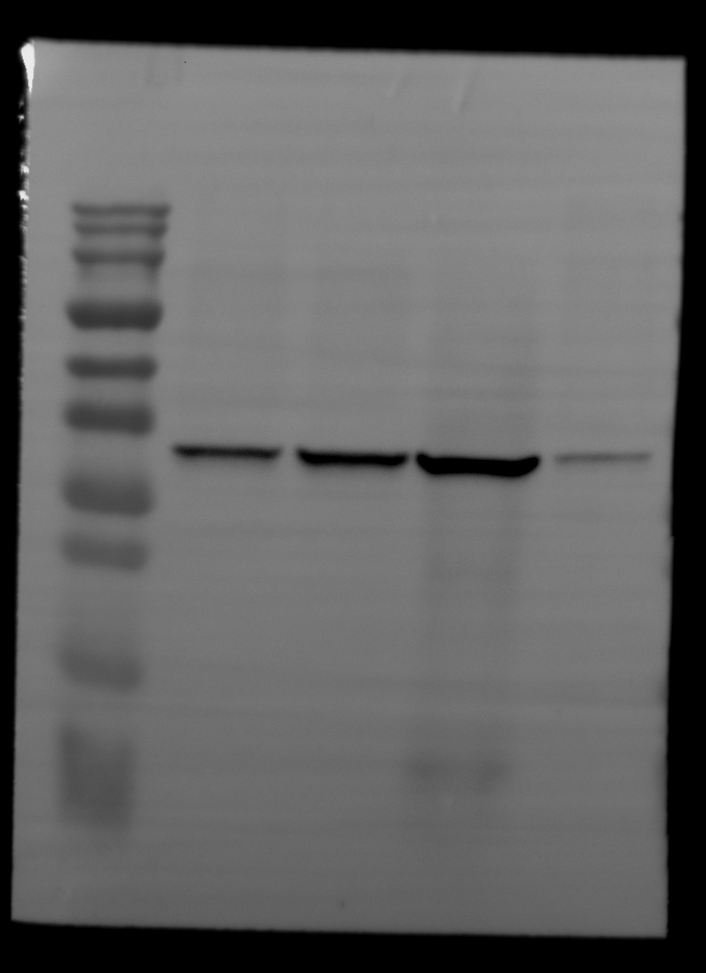


**Supplemental fig. 2** The original, uncropped Western blot images of IL-1β


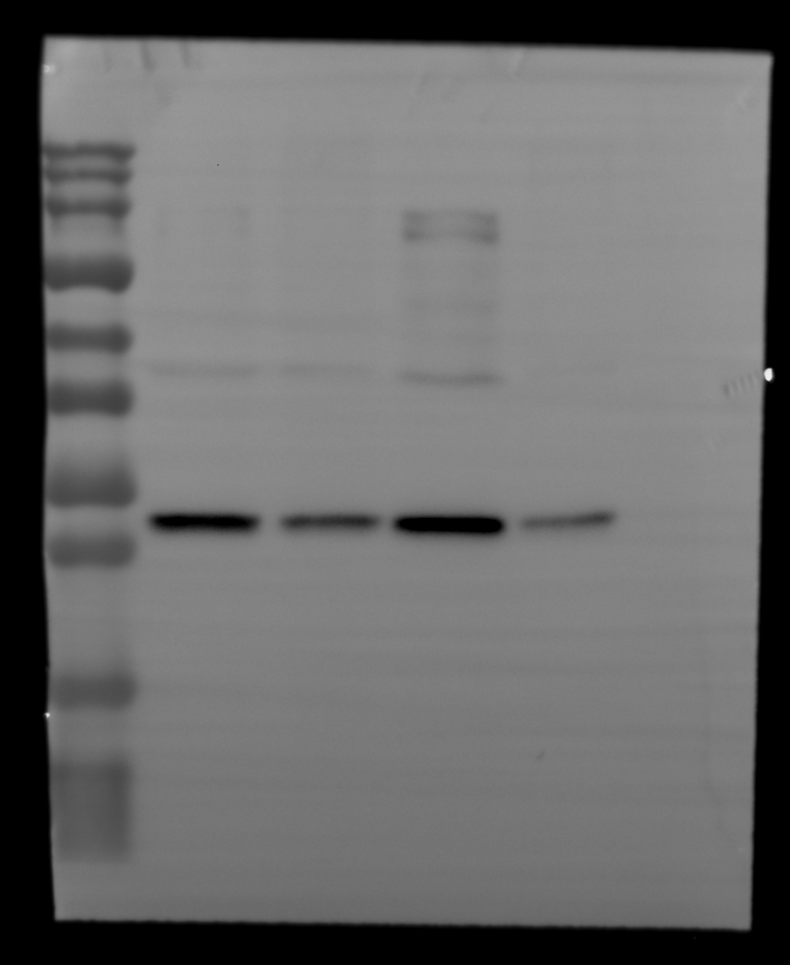


**Supplemental fig. 3** The original, uncropped Western blot images of IL-6


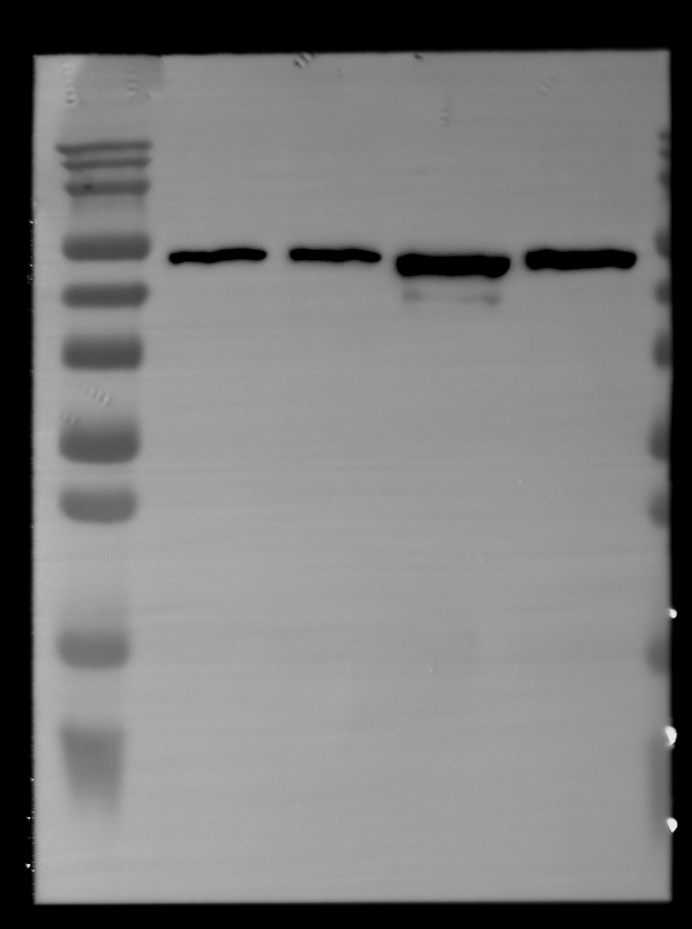


**Supplemental fig. 4** The original, uncropped Western blot images of TNF-α


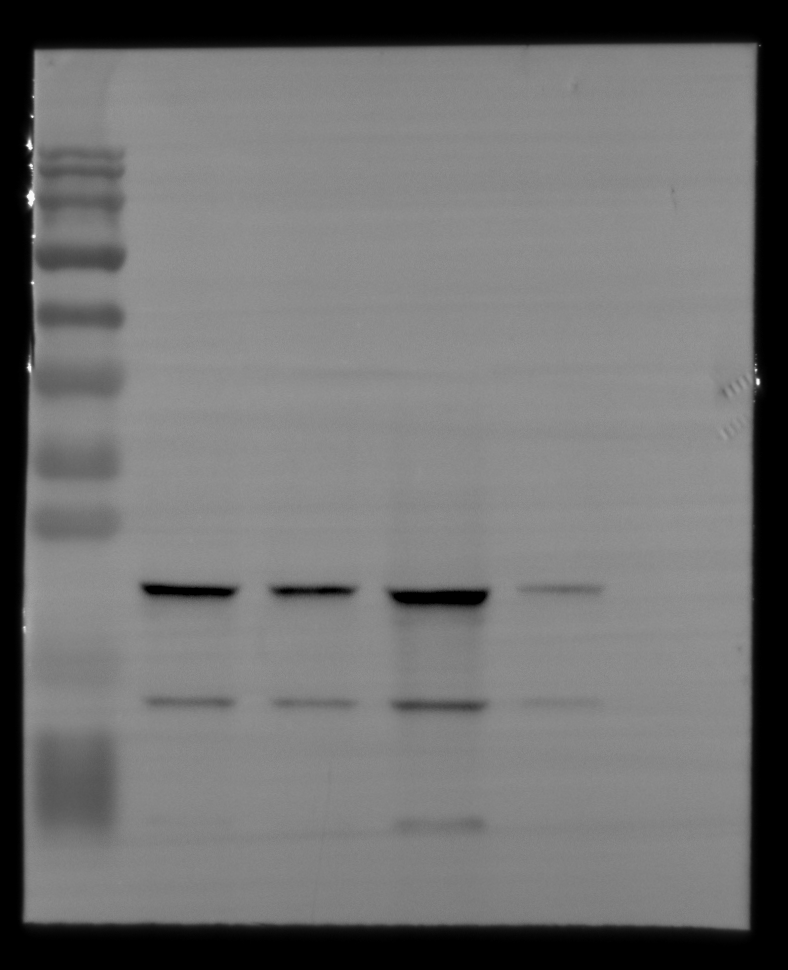


**Supplemental fig. 5** The original, uncropped Western blot images of S100A9


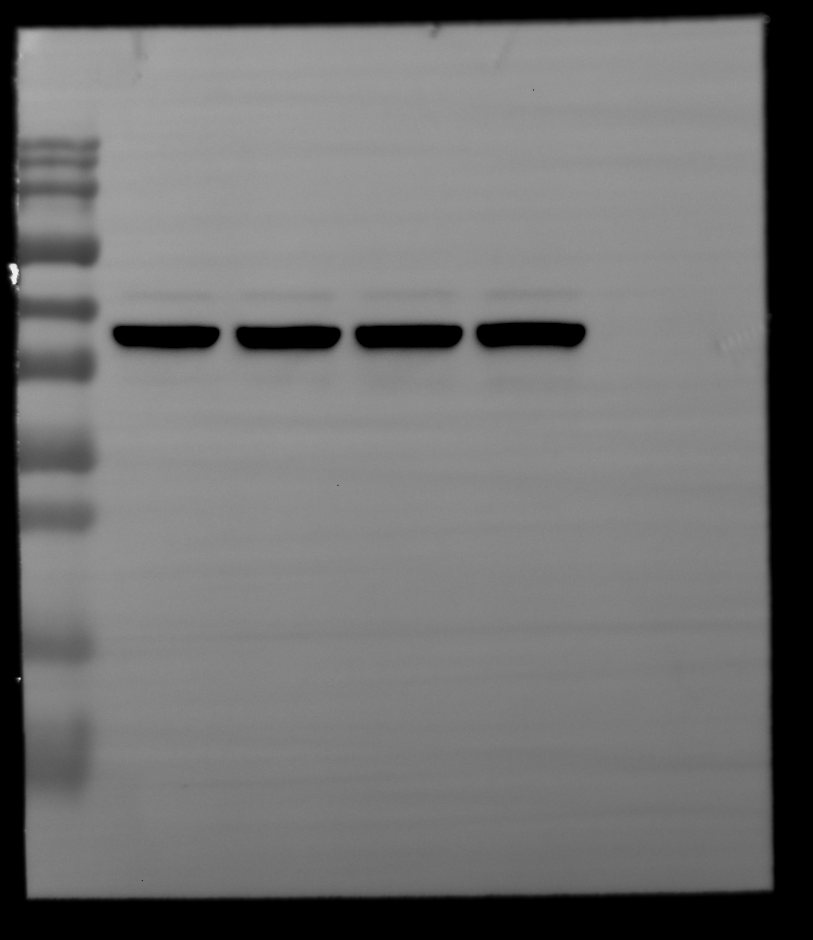


**Supplemental fig. 6** The original, uncropped Western blot images of ACTIN1


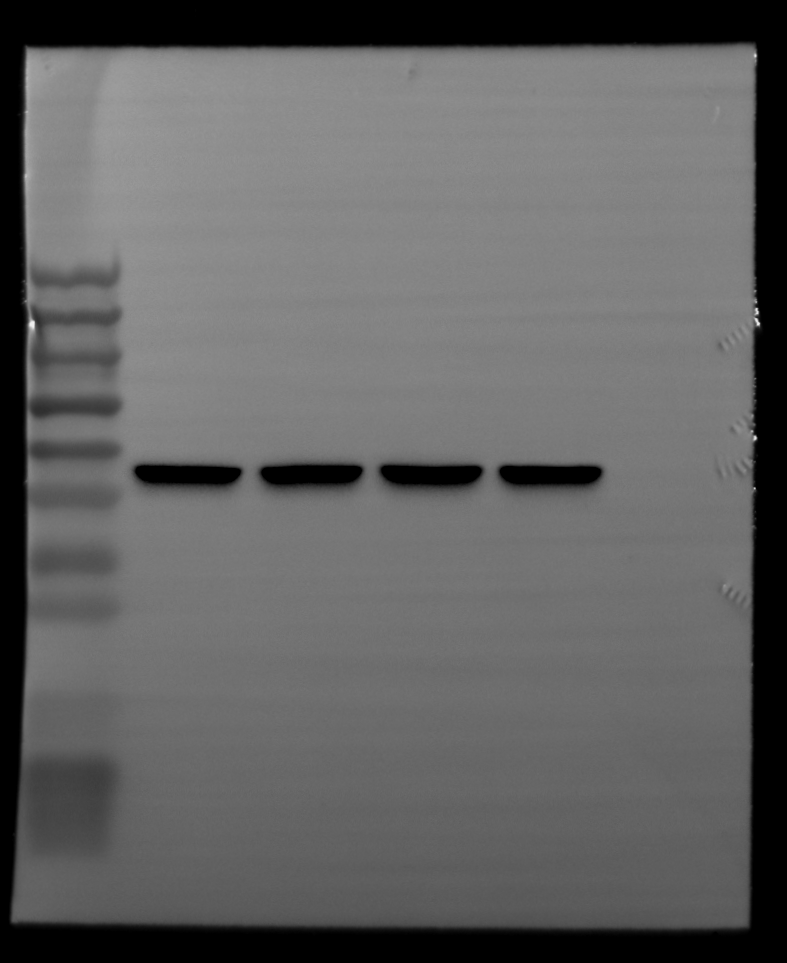


**Supplemental fig. 7** The original, uncropped Western blot images of ACTIN2

**Supplemental table 1 | Cell numbers of each cluster**

| **Groups** | B cells | Endothelial | Fast muscle | Fibroblasts | Macrophages | MuSCs | Neutrophils | T cells |
| --- | --- | --- | --- | --- | --- | --- | --- | --- |
| CON | 0 | 1055 | 99 | 1682 | 856 | 217 | 47 | 437 |
| CTX | 41 | 1881 | 488 | 1419 | 3477 | 277 | 875 | 486 |
